# Supplementary material for: Incidence of Severe COVID-19 Outcomes and Immunization Rates in Apulian Individuals with Inflammatory Bowel Disease: A Retrospective Cohort Study
Source: Vaccines (Basel). 2024 Aug 2;12(8):881. doi: 10.3390/vaccines12080881 (PMC11359773; doi:10.3390/vaccines12080881)

Supplementary Materials

Table S1. SARS-CoV-2 Incidences rate, stratified per group, age class and sex.

Variable	Control group	Individuals with IBD	Total	IRR (95%CI)
Sex				
• female	1.46 (1.36-1.58)	1.39 (1.22-1.60)	1.45 (1.35-1.55)	0.95 (0.81-1.12)
• male	1.26 (1.12-1.36)	1.24(1.08-1.41)	1.25 (1.17-1.34)	0.98 (0.84-1.14)
Age class				
• 18-49	1.58 (1.47-1.70)	1.53 (1.34-1.74)	1.57 (1.47-1.67)	0.97 (0.83-1.12)
• 50-64	1.32 (1.19-1.46)	1.28 (1.06-1.53)	1.31 (1.20-1.43)	0.97 (0.78-1.19)
• 65+	0.96 (0.84-1.09)	0.92 (0.74-1.16)	0.94 (0.85-1.06)	0.97 (0.74-1.26)

Table S2. Multivariate Cox semiparametric regression analyses of the risk predictors of OS.

Determinant	Basal routine	
	aHR (95%CI)	p-value
Group variable (Individuals with IBD vs. General population)	0.95 (0.84-1.07)	0.367
Age (yrs)	0.99 (0.98-0.99)	<0.0001
Sex (male vs. female)	0.87 (0.79-0.96)	0.004
Comorbidities		
• 1 vs. none	1.03 (0.93-1.16)	0.548
• ≥2 vs. none	1.00 (0.86-1.17)	0.979
Basal vaccination routine*	-	-

Goodness-of-fit p-value=0.616

***proportionality not reached**

Table S3. Characteristic of patients with IBD, per disease (Crohn vs. ulcerative colitis).

Variable	Crohn's disease (n=662)	Ulcerative colitis (n=367)	Total (n=1,029)	p-value
Male; n (%)	353 (53.3)	211 (57.5)	564 (54.8)	0.198
Age at the start of pandemic; mean±SD (range)	48.8±1 (18-91)	54.4±17.0 (18-91)	50.8±16.8 (18-96)	<0.0001
Age class; n (%)<br>• 18-49<br>• 50-64<br>• 65+	 349 (52.7)<br>185 (28.0)<br>128 (19.3)	 146 (39.8)<br>105 (28.6)<br>116 (31.6)	 495 (48.1)<br>290 (28.2)<br>244 (23.7)	<0.0001
COVID19 incidence rate (x100 persons-month)	1.30 (95%CI=1.15-1.50)	1.32 (95%CI=1.13-1.55)	1.31 (95%CI=1.28-1.43)	IRR=1.02; 95%CI=0.83-1.24); p=0.425
COVID19 hospitalization; n (%)	9 (1.4)	7 (1.9)	16 (1.6)	0.496
Case-fatality rate; n (%)	0 (0.0)	1 (0.3)	1 (0.1)	0.357
Vaccine coverage; n (%; 95%CI)<br>• basal routine<br>• first booster<br>• second booster	 597 (92.0; 89.6-94.0)<br>514 (79.8; 76.5-82.8)<br>73 (11.3; 9.0-14.0)	 321 (90.4; 86.9-93.3)<br>273 (78.0; 73.2-82.2)<br>47 (13.7; 10.2-17.8)	 918 (91.4; 89.5-93.1)<br>787 (79.2; 76.5-81.7)<br>120 (12.2; 10.1-14.4)	 0.397<br>0.452<br>0.279

Table S4. Characteristics of hospitalized and dead individuals with IBD, per disease.

Variable	Crohn's disease	Ulcerative colitis	Total IBD
**Hospitalization**			
Age; mean±SD (range)	58.4±13.2 (42-82)	68.3±15.2 (37-81)	62.8±14.5 (37-82)
Male; n (%)	4 (44.4)	4 (57.1)	8 (50.0)
Age class; n (%)			
• 18-49	3 (33.3)	1 (14.3)	4 (25.0)
• 50-64	4 (44.4)	1 (14.3)	5 (31.3)
• 65+	2 (22.3)	5 (71.4)	7 (43.7)
COVID-19 basal routine; n (%)	8 (88.9)	5 (71.4)	13 (81.3)
**Death**			
Age; mean±SD (range)	-	81	81
Male; n (%)	-	0 (0.0)	0 (0.0)
Age class; n (%)	-		
• 18-49		0 (0.0)	0 (0.0)
• 50-64		0 (0.0)	0 (0.0)
• 65+		1 (100.0)	1 (100.0)
COVID-19 basal routine; n (%)	-	0 (0.0)	0 (0.0)

Table S5. Multivariate logistic regression models of COVID-19 vaccine uptake, per dose.

Determinant	Basal routine		First booster		Second booster	
	aOR (95%CI)	p-value	aOR (95%CI)	p-value	aOR (95%CI)	p-value
Crohn’s disease vs. Ulcerative colitis	0.71 (0.44-1.13)	0.145	0.76 (0.55-1.07)	0.118	0.81 (0.52-1.25)	0.339
Age (yrs)	1.03 (1.01-1.04)	<0.0001	1.03 (1.02-1.04)	<0.0001	1.07 (1.06-1.09)	<0.0001
Sex (male vs. female)	1.05 (0.67-1.64)	0.844	0.98 (0.72-1.35)	0.922	1.49 (0.98-2.28)	0.064
Comorbidities						
• 1 vs. none	1.04 (0.58-1.85)	0.905	1.35 (0.91-2.00)	0.134	0.99 (0.55-1.76)	0.962
• ≥2 vs. none	1.24 (0.57-2.67)	0.591	2.01 (1.17-4.46)	0.011	1.76 (0.97-3.17)	0.061
		Goodness-of-fit p-value=0.673		Goodness-of-fit p-value=0.623		Goodness-of-fit p-value=0.081

Figure S1. Cumulative incidence of documented SARS-CoV-2 infection, per disease (Crohn vs. ulcerative colitis).

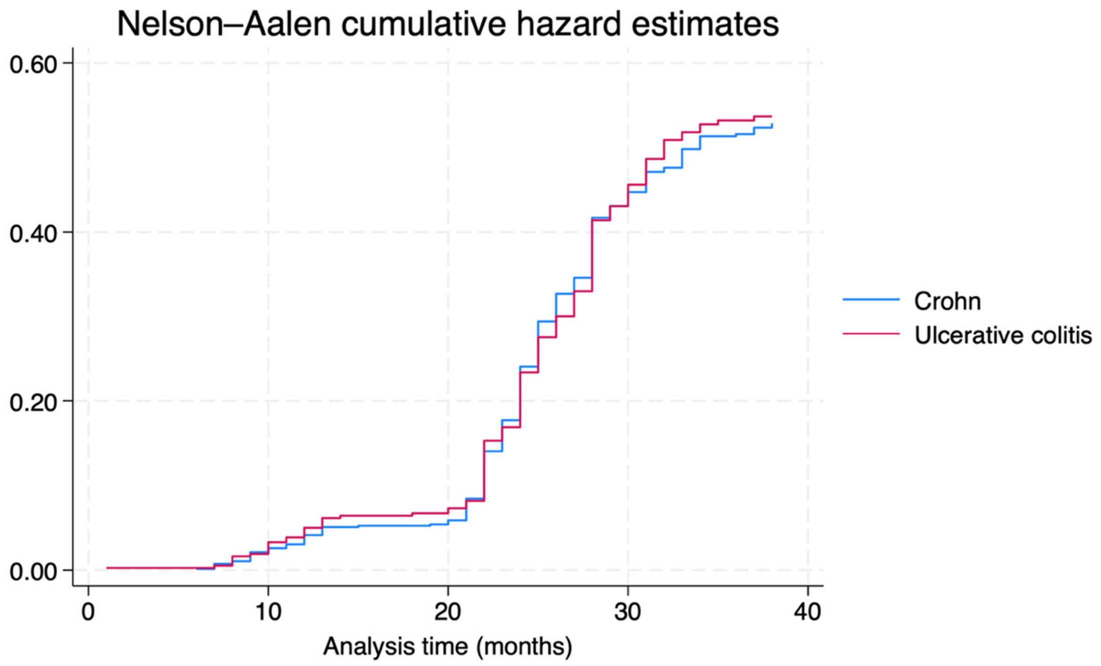

Log-rank p-value=0.909

Figure S2. COVID-19 vaccine coverage, per group (individuals with IBD vs. general population).

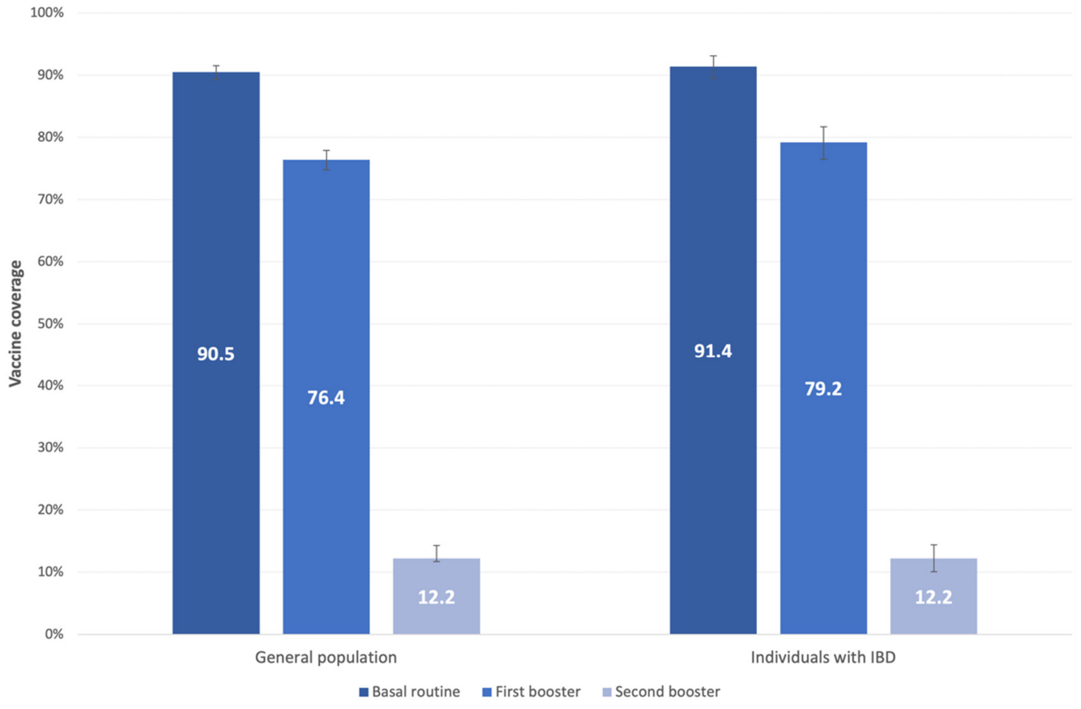

Supplement: Supplementary file 1 [file vaccines-12-00881-s001.zip › vaccines-3093242-supplementary.pdf]
